# Molecular Oncodiagnostics in Precision Oncology: Integrating Tumor Transcriptomics, Patient Pharmacogenetics, and Ex Vivo Chemoresistance Testing to Improve Individual Chemotherapy Response

**DOI:** 10.3390/jpm16040176

**Published:** 2026-03-24

**Authors:** Dario Rusciano

**Affiliations:** Independent Researcher, 57125 Livorno, Italy; drusciano55@gmail.com

**Keywords:** precision oncology, tumor transcriptomics, pharmacogenetics, ex vivo chemoresistance testing, functional precision medicine

## Abstract

**Background**: Precision oncology has traditionally relied on genomic biomarkers to guide therapy selection; however, static molecular profiling often fails to predict real-world responses to cytotoxic chemotherapy. Increasing evidence suggests that treatment outcomes are determined by the interaction between tumor-intrinsic biology and host-specific pharmacology. Functional ex vivo platforms, including patient-derived organoids and tumor slice cultures, provide a complementary phenotypic readout of drug sensitivity that reflects tumor architecture and microenvironmental interactions. **Methods**: This narrative review integrates recent experimental, translational, and clinical evidence on molecular oncodiagnostics combining tumor transcriptomics, germline pharmacogenetics, and ex vivo drug sensitivity testing. Relevant literature was identified through targeted searches of major biomedical databases, focusing on studies describing multi-omic predictive models, functional precision oncology platforms, and patient-derived tumor models. **Results**: Converging data indicate that integrated oncodiagnostic strategies can improve prediction of chemotherapy response beyond genomics-only approaches. Transcriptomic profiling captures dynamic pathway activity and resistance programs, pharmacogenetic testing informs host-specific toxicity and dosing constraints, and ex vivo assays enable direct phenotypic validation of drug efficacy. Together, these complementary approaches provide a biologically grounded framework for individualized therapy selection. **Conclusions**: The convergence of molecular profiling and functional phenotyping represents an emerging paradigm in precision oncology. Integrating multi-omic and functional data may enhance treatment prediction and reduce ineffective therapy, although prospective validation and standardization remain necessary for routine clinical implementation.

## 1. Introduction

### 1.1. The Unmet Need for Individualized Chemotherapy

Despite substantial progress in precision oncology, conventional chemotherapeutic regimens remain associated with pronounced inter-patient variability in both therapeutic efficacy and treatment-limiting toxicity. Patients with comparable tumor histology, disease stage, and even shared molecular driver alterations frequently display markedly divergent clinical responses when exposed to identical cytotoxic protocols. This variability arises from a complex interplay between tumor biology, host pharmacokinetics and pharmacodynamics, and microenvironmental influences. Many of these factors remain poorly captured by current clinical and molecular stratification systems [1,2].

Historically, oncology treatment selection has relied on histopathological classification and, more recently, on the identification of actionable genomic alterations. These approaches have improved outcomes for selected patient subgroups. However, they remain limited in predicting responses to most cytotoxic agents, which exert complex and context-dependent effects on tumor cells and surrounding stromal and immune compartments [3,4]. Genotype-centric models fail to account for transcriptional plasticity, pathway redundancy, metabolic adaptation, and stress-response programs that critically shape chemosensitivity and acquired resistance [1,2].

The clinical implications of these limitations are substantial. Primary resistance to chemotherapy exposes patients to unnecessary toxicity without therapeutic benefit, while unpredictable adverse drug reactions frequently necessitate dose reductions, treatment delays, or premature discontinuation, ultimately compromising oncologic outcomes and quality of life [5,6]. Together, these challenges underscore an urgent unmet need for oncodiagnostic strategies capable of prospectively identifying chemotherapeutic regimens that maximize efficacy while minimizing toxicity at the individual patient level.

### 1.2. From Precision Oncology to Functional Precision Oncology

Precision oncology has traditionally been driven by a mutation-centric paradigm aimed at matching oncogenic driver alterations with targeted therapies. However, it is increasingly evident that static genomic information alone provides an incomplete representation of tumor behavior, particularly in the context of cytotoxic chemotherapy, where treatment response is influenced by dynamic cellular states rather than single genetic lesions [4,7]. Consequently, the field is undergoing a conceptual transition toward functional precision oncology, which integrates multi-layer molecular data to better predict actual drug response.

Within this framework, tumor transcriptomics has emerged as a powerful approach for capturing biologically relevant cellular programs, including pathway activation, DNA damage response capacity, metabolic reprogramming, and drug transport mechanisms. Accumulating evidence indicates that gene expression-based signatures can outperform genomic alterations in predicting sensitivity or resistance to multiple chemotherapeutic classes across diverse solid tumors [3,8,9]. Transcriptome-guided therapeutic alignment has demonstrated particular promise in treatment-refractory malignancies, highlighting its potential clinical utility beyond genomics-only approaches [9,10].

In parallel, advances in germline pharmacogenetics have clarified the role of inherited variants in drug-metabolizing enzymes, transporters, and DNA repair pathways as major determinants of chemotherapy-related toxicity and, in some settings, therapeutic efficacy. Contemporary clinical guidelines increasingly endorse genotype-guided dosing or drug selection for widely used agents such as fluoropyrimidines, thiopurines, and irinotecan [11,12,13]. Nevertheless, pharmacogenetic data are still rarely integrated with tumor-specific molecular profiles or functional response assays in routine oncology practice.

Collectively, these converging insights support the need for an integrated oncodiagnostic paradigm that simultaneously incorporates tumor transcriptomic profiling, patient germline pharmacogenetics, and functional drug response testing. Such an approach holds the potential to bridge the gap between molecular characterization and real-world therapeutic outcomes, moving beyond predictive biomarkers toward actionable, patient-specific treatment strategies [2,14].

### 1.3. Aim and Scope of the Review

The aim of this review is to critically examine recent evidence supporting the integration of tumor transcriptomics, patient germline pharmacogenetics, and functional chemosensitivity testing as complementary pillars of next-generation molecular oncodiagnostics. Specifically, we survey contemporary studies linking (i) tumor gene expression profiles, (ii) inherited pharmacogenetic variants, and (iii) differential therapeutic response and toxicity to standard chemotherapeutic protocols across both solid and hematological malignancies.

Building on this evidence base, we propose a conceptual framework for integrated molecular oncodiagnostics that moves beyond static biomarkers toward a dynamic, patient- and tumor-specific model for predicting chemotherapy response. By synthesizing advances from precision oncology, pharmacogenomics, and functional testing platforms, this review aims to highlight both the opportunities and the translational challenges associated with implementing truly individualized chemotherapy in clinical practice.

**Figure 1:**
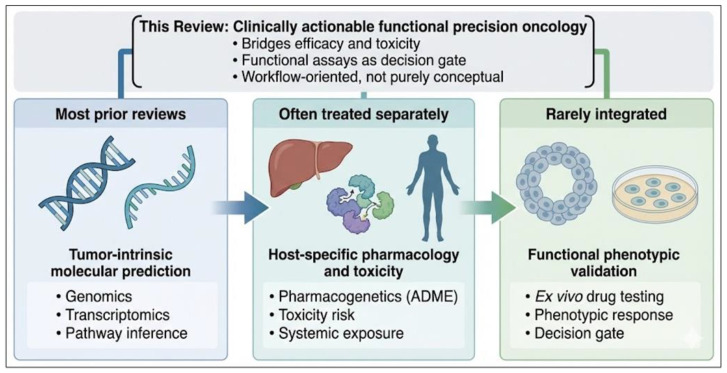
Conceptual positioning of this review relative to existing precision oncology approaches. The figure illustrates how the proposed framework extends beyond tumor-intrinsic molecular prediction by integrating host-specific pharmacology and toxicity with functional phenotypic validation, thereby framing ex vivo drug testing as a decision gate within a clinically actionable, workflow-oriented model of functional precision oncology.

Several recent reviews have addressed individual components of precision oncology, including tumor genomics, transcriptomics, pharmacogenomics, artificial intelligence, and functional drug testing. In contrast, the present review positions these elements within a unified translational framework that links tumor-intrinsic efficacy prediction with host-specific toxicity and tolerability assessment. Importantly, the framework proposed here does not represent a new experimental platform but rather a conceptual integration of diagnostic layers that are often discussed separately in the precision oncology literature. By combining tumor transcriptomic state analysis, germline pharmacogenetic profiling, and ex vivo drug-response testing within a single workflow, the model aims to bridge molecular prediction and functional validation in chemotherapy selection. Rather than treating functional assays as purely correlative tools, this framework highlights ex vivo drug sensitivity testing as a biological decision gate capable of confirming or refuting molecular predictions. In doing so, the review outlines a clinically oriented workflow that clarifies the complementary roles, maturity, and limitations of molecular profiling, computational modeling, and functional validation within contemporary precision oncology (Figure 1).

### 1.4. Literature Search Strategy

The literature considered in this review was identified through targeted searches of PubMed/MEDLINE, Scopus, and Web of Science, covering publications from database inception through February 2026. Search terms included combinations of precision oncology, tumor transcriptomics, pharmacogenetics, multi-omics integration, ex vivo drug sensitivity testing, patient-derived organoids, and functional precision oncology.

Titles and abstracts were screened for relevance to molecular predictors of chemotherapy response, host pharmacogenetic determinants of drug toxicity, and functional ex vivo platforms capable of evaluating patient-specific tumor drug sensitivity. Priority was given to original research articles, translational studies, and mechanistically informative reviews addressing the integration of tumor molecular profiling with functional drug response assays.

Particular emphasis was placed on recent publications (2020–2025) describing advances in multi-omic predictive modeling, AI-supported oncodiagnostics, and patient-derived tumor models used for phenotypic drug testing.

The final selection of references was curated to support a mechanism-oriented narrative illustrating the conceptual transition from static molecular profiling toward integrated molecular–functional frameworks for individualized chemotherapy selection.

## 2. Molecular Oncodiagnostics: Conceptual and Technical Foundations

### 2.1. Definition and Components

Molecular oncodiagnostics can be defined as the integrated use of tumor-derived molecular information and patient-specific biological data to inform therapeutic decision-making across the cancer care continuum, reflecting the broader shift toward comprehensive diagnostic frameworks that combine multiple molecular layers within precision oncology workflows [15]. Unlike traditional diagnostic paradigms that focus primarily on histopathology and limited genomic markers, molecular oncodiagnostics aims to capture the dynamic biological determinants of drug response, resistance, and toxicity at both the tumor and host levels [2,16].

A fundamental distinction within this framework lies between tumor genomics and tumor transcriptomics. Tumor genomics focuses on the identification of somatic DNA alterations—such as point mutations, copy number variations, and structural rearrangements—that drive oncogenesis and can serve as therapeutic targets. While genomic profiling has enabled the development of targeted and tumor-specific therapies, it provides a largely static snapshot of cancer biology and often fails to predict response to cytotoxic chemotherapy [4,7]. In contrast, tumor transcriptomics captures gene expression programs that reflect active signaling pathways, cellular stress responses, metabolic states, and microenvironmental interactions, offering a more dynamic and functionally relevant view of tumor behavior [3,8].

An equally critical distinction exists between somatic tumor alterations and germline pharmacogenetics. Somatic alterations arise within cancer cells and influence tumor sensitivity or resistance to therapy, whereas germline pharmacogenetic variants are inherited and affect drug absorption, distribution, metabolism, and elimination, as well as susceptibility to treatment-related toxicity. Germline variants in genes encoding drug-metabolizing enzymes, transporters, and DNA repair proteins have been shown to significantly modulate chemotherapy tolerance and, in some cases, efficacy [11,12,13]. Molecular oncodiagnostics therefore extends beyond tumor-centric profiling to incorporate host-specific determinants that shape real-world treatment outcomes.

Within precision medicine workflows, molecular oncodiagnostics occupies a central integrative role. It bridges upstream molecular characterization (genomics, transcriptomics, pharmacogenetics) with downstream therapeutic selection, dose optimization, and toxicity management. Importantly, it also provides the conceptual foundation for functional precision oncology approaches, in which molecular data are complemented by ex vivo or in silico models to directly interrogate drug response [14,16].

### 2.2. Enabling Technologies

The clinical translation of molecular oncodiagnostics has been driven by rapid advances in high-throughput molecular technologies and next-generation sequencing platforms, which enable comprehensive characterization of genomic and transcriptomic alterations relevant to cancer diagnosis and treatment selection [3,9,17]. Among these approaches, bulk RNA sequencing (RNA-seq) remains the most widely used method for transcriptomic profiling, enabling the identification of gene expression signatures associated with prognosis and therapeutic response. Targeted RNA panels focusing on predefined gene sets linked to specific pathways or drug classes have emerged as cost-effective and clinically scalable alternatives, particularly when tissue availability is limited [10].

More recently, single-cell transcriptomic technologies have provided unprecedented resolution into intratumoral heterogeneity and cell-state-specific drug resistance mechanisms. Single-cell RNA-seq has revealed coexisting sensitive and resistant subpopulations within the same tumor and has elucidated the role of immune and stromal compartments in shaping chemotherapy response [18,19,20]. Although currently limited by cost, complexity, and data interpretation challenges, these approaches are increasingly informing biomarker discovery and translational research pipelines.

In parallel, germline pharmacogenetic testing platforms have evolved from single-gene assays to multiplex next-generation sequencing panels and high-density SNP arrays. These platforms enable simultaneous assessment of multiple pharmacogenes relevant to oncology prescribing, supporting genotype-guided dosing and drug selection for commonly used chemotherapeutic agents [11,13]. Despite strong evidence and growing guideline support, the integration of pharmacogenetic testing into routine oncology practice remains inconsistent, in part due to logistical and interpretive barriers [21].

The analytical complexity of molecular oncodiagnostics necessitates robust bioinformatic pipelines capable of integrating heterogeneous data types, including genomic variants, transcriptomic signatures, and clinical annotations. Challenges persist in data normalization, cross-platform reproducibility, and clinical reporting, particularly when translating probabilistic molecular signals into actionable treatment recommendations [22,23]. Standardization of analytical workflows and reporting frameworks remains a critical prerequisite for broader clinical adoption.

### 2.3. Clinical Implementation Models

Clinical implementation of molecular oncodiagnostics varies widely across healthcare systems, reflecting differences in infrastructure, regulatory environments, and resource availability. Centralized molecular diagnostic models, often based in academic or reference laboratories, offer access to advanced technologies, specialized bioinformatics expertise, and standardized quality control. However, they may be limited by longer turnaround times and reduced flexibility for iterative testing [2,16].

In contrast, decentralized or hybrid models aim to embed molecular testing capabilities within regional cancer centers, facilitating closer integration with clinical workflows and enabling rapid feedback between diagnostic results and therapeutic decisions. While potentially more responsive, these models face challenges related to scalability, cost, and maintenance of analytical consistency across sites [21,22].

Across both models, molecular tumor boards (MTBs) have emerged as a key implementation strategy for interpreting complex molecular data and translating them into individualized treatment plans. MTBs bring together oncologists, pathologists, molecular biologists, geneticists, and bioinformaticians to contextualize genomic, transcriptomic, and pharmacogenetic findings within the clinical scenario [4,16]. Increasingly, MTBs are being supported by digital decision-support systems and artificial intelligence–driven tools designed to synthesize rapidly evolving evidence bases and assist clinical prioritization [23].

Together, these implementation models illustrate that molecular oncodiagnostics is not merely a technological innovation but a systemic transformation of oncology practice. Its successful integration requires coordinated advances in laboratory infrastructure, data analytics, multidisciplinary collaboration, and clinical governance.

## 3. Tumor Transcriptomics as a Predictor of Chemotherapy Response

### 3.1. Biological Rationale

Tumor gene expression profiles provide a functional readout of the biological state of cancer cells, integrating the effects of genetic alterations, epigenetic regulation, microenvironmental signals, and therapy-induced stress responses. Unlike genomic alterations, which represent relatively static features of the tumor, transcriptomic patterns dynamically reflect pathway activity and cellular behavior at the time of sampling, making them particularly relevant for predicting chemotherapy response [3,8].

One of the most consistent transcriptomic determinants of chemosensitivity is cellular proliferation. Expression signatures enriched for cell-cycle progression and replication machinery correlate with enhanced sensitivity to DNA-damaging agents and antimitotic drugs, reflecting the preferential targeting of rapidly dividing cells by many cytotoxic compounds [3].

DNA damage response and repair capacity represents another transcriptionally regulated axis influencing chemotherapy efficacy. Reduced expression of genes involved in homologous recombination, nucleotide excision repair, and replication fork stability has been associated with increased sensitivity to platinum compounds and other genotoxic agents, whereas transcriptional upregulation of repair pathways contributes to both intrinsic and acquired resistance [8,9].

Transcriptomic control of drug transport and metabolism further modulates intracellular drug exposure. Differential expression of solute carriers, ATP-binding cassette transporters, and detoxifying enzymes can markedly influence chemotherapy accumulation and clearance within tumor cells, independently of somatic mutations [10].

The execution of cytotoxic effects also depends on apoptotic competence, which is largely transcriptionally governed. Expression levels of pro-apoptotic and anti-apoptotic regulators, stress response pathways, and autophagy-related genes have been repeatedly linked to differential responses to chemotherapy across solid and hematologic malignancies [9].

Finally, tumor transcriptomes encode signals arising from immune and stromal interactions, which increasingly appear to modulate chemotherapy efficacy. Importantly, the cellular composition of solid tumors extends beyond malignant epithelial cells and includes a heterogeneous tumor microenvironment composed of cancer-associated fibroblasts, endothelial cells, pericytes, and diverse immune populations such as tumor-associated macrophages, lymphocytes, dendritic cells, and myeloid-derived suppressor cells. These cellular compartments collectively contribute to the structural and functional architecture of the tumor bulk and regulate key processes including extracellular matrix remodeling, angiogenesis, immune surveillance, and drug penetration. Consequently, bulk transcriptomic profiles frequently capture signals derived not only from malignant cells but also from stromal and immune compartments, reflecting the integrated behavior of the tumor ecosystem. Expression patterns reflecting immune infiltration, inflammatory signaling, and fibroblast activity can therefore influence drug distribution, immunogenic cell death, and therapy-induced immune modulation, contributing to inter-patient variability in treatment response [18,19,24].

### 3.2. Transcriptomic Signatures Associated with Drug Sensitivity and Resistance

A growing body of evidence supports the association between specific gene expression programs and response to distinct classes of chemotherapeutic agents. For platinum-based compounds, transcriptomic predictors consistently highlight DNA repair proficiency, oxidative stress responses, and cell-cycle control. In ovarian, lung, and colorectal cancers, expression profiles indicative of impaired homologous recombination or heightened replication stress have been associated with improved platinum sensitivity, whereas adaptive transcriptional activation of repair and detoxification pathways underlies resistance [8,9].

Anthracycline responsiveness has been linked to transcriptional programs reflecting proliferative activity, topoisomerase II expression, and oxidative stress handling. In breast cancer, multigene expression signatures have demonstrated predictive value for anthracycline benefit beyond traditional clinicopathological parameters [3].

For taxanes, transcriptomic correlates of sensitivity involve genes regulating microtubule dynamics, mitotic checkpoint integrity, and epithelial–mesenchymal transition. Tumors characterized by mesenchymal-like transcriptional states or altered spindle assembly checkpoint signaling frequently display reduced taxane responsiveness, a pattern observed across breast, lung, and ovarian malignancies [10].

Antimetabolite sensitivity is influenced by expression of nucleotide biosynthesis pathways, DNA replication machinery, and metabolic adaptability. In colorectal cancer and hematologic malignancies, transcriptional profiles indicating high replicative stress and limited metabolic plasticity correlate with enhanced response, whereas metabolic reprogramming supports resistance [9].

Emerging data also support transcriptomic predictors of response to targeted cytotoxic therapies, including antibody–drug conjugates. In these settings, expression of target antigens, intracellular trafficking pathways, and immune effector signatures jointly determine therapeutic efficacy, reinforcing the value of composite expression-based models over single-gene markers [19,20].

### 3.3. Clinical Assays and Commercial Platforms

Transcriptomic insights have been translated into multigene expression assays used in clinical decision-making, particularly in early-stage breast cancer. Platforms such as Oncotype DX and MammaPrint exemplify how composite gene expression signatures can inform chemotherapy use by estimating both recurrence risk and likelihood of treatment benefit [3].

Beyond breast cancer, transcriptome-guided approaches are increasingly being explored to inform chemotherapy selection in gastrointestinal, lung, and hematologic malignancies. Recent transcriptomic precision oncology initiatives aim to match patients to therapies based on global expression patterns rather than isolated genomic alterations, expanding therapeutic options for patients lacking actionable mutations [8,9].

The principal strengths of current transcriptomic predictors lie in their ability to integrate multiple biological processes and provide probabilistic estimates of benefit. However, limitations remain, including variability in assay platforms, bioinformatic pipelines, and the need for robust prospective validation across diverse clinical settings [10].

### 3.4. Limitations and Unresolved Challenges

Several challenges continue to constrain the clinical implementation of transcriptomic predictors. Tumor heterogeneity represents a major limitation, as bulk expression profiles derived from single biopsies may not capture spatially distinct subclones with divergent drug sensitivities. Single-cell and spatial transcriptomic studies increasingly reveal intratumoral diversity that can undermine the predictive accuracy of bulk assays [18,20].

The temporal plasticity of the transcriptome further complicates prediction, as gene expression programs evolve under therapeutic pressure. Pre-treatment transcriptomic profiles may therefore fail to fully anticipate response to subsequent treatment lines, particularly in the context of adaptive resistance mechanisms [19].

Finally, the influence of the tumor microenvironment challenges tumor-centric interpretation of transcriptomic data. Stromal and immune components contribute substantially to bulk expression signatures, complicating attribution of predictive signals to malignant cells alone. While this complexity reflects biological reality, it underscores the need for refined analytical strategies and integrative models to improve clinical utility [18,19].

## 4. Patient Pharmacogenetics in Oncology

Germline pharmacogenetics (PGx) investigates how inherited genetic variation influences individual responses to anticancer drugs, with particular emphasis on predicting toxicity and informing dose optimization. In contrast to tumor-derived molecular profiling, which captures cancer-specific vulnerabilities, pharmacogenetics reflects stable host characteristics that affect drug handling independently of tumor biology. As such, PGx represents a complementary pillar of molecular oncodiagnostics, addressing a critical dimension of inter-patient variability that cannot be resolved through tumor profiling alone [11,13].

### 4.1. Germline Pharmacogenetics: Principles and Relevance

Germline variants are inherited and present in all somatic cells, shaping drug disposition and host susceptibility to adverse effects regardless of tumor type or molecular subtype. The core biological basis of PGx lies in inherited differences in genes involved in absorption, distribution, metabolism, and excretion (ADME) of drugs. Variants affecting enzymatic activity, transporter expression, or cofactor availability can profoundly alter systemic drug exposure, leading to either excessive toxicity or subtherapeutic dosing [25,26].

One of the most clinically impactful applications of PGx is the prediction of drug-induced toxicity. Many chemotherapeutic agents have narrow therapeutic indices, and standard body surface area-based dosing fails to account for genetically determined differences in drug clearance. PGx testing enables prospective identification of patients at high risk for severe adverse drug reactions (ADRs), allowing dose adjustment or alternative therapy selection before treatment initiation [5,11].

Closely related is the prevention of dose-limiting adverse events, which remain a major cause of treatment interruption and long-term morbidity. Severe myelosuppression, mucositis, diarrhea, and hepatotoxicity frequently necessitate dose reductions or early discontinuation, compromising therapeutic efficacy. By identifying patients predisposed to such toxicities, pharmacogenetic testing supports safer treatment delivery and helps preserve intended dose intensity [6,27].

### 4.2. Key Pharmacogenetic Pathways in Chemotherapy

Several pharmacogenetic pathways have achieved robust clinical validation and are directly relevant to commonly used chemotherapeutic agents. Among enzymatic pathways, Dihydropyrimidine Dehydrogenase (DPYD) represents the most established example. Pathogenic variants in DPYD lead to partial or complete deficiency of DPYD, the rate-limiting enzyme in fluoropyrimidine catabolism. Carriers of reduced-function alleles are at markedly increased risk of life-threatening toxicity following treatment with 5-fluorouracil or capecitabine, even at standard doses [11,13].

Similarly, Thiopurine S-Methyltransferase (TPMT) and Nudix Hydrolase 15 (NUDT15) variants critically influence thiopurine metabolism. Deficiency in either enzyme results in excessive accumulation of cytotoxic thioguanine nucleotides, predisposing patients to severe and sometimes fatal myelosuppression. Genotype-guided dose adjustment for thiopurines is now widely accepted in both hematologic malignancies and solid tumor protocols incorporating these agents [12,25].

The Uridine Diphosphate Glucuronosyltransferase Family 1 Member A1 (UGT1A1) pathway is central to irinotecan metabolism. Reduced-function UGT1A1 alleles impair glucuronidation of SN-38, the active irinotecan metabolite, increasing the risk of neutropenia and severe diarrhea. Pharmacogenetic testing allows identification of high-risk individuals who may benefit from initial dose reduction or alternative regimens [11,13].

Beyond metabolic enzymes, drug transporters play a critical role in systemic and tissue-level drug exposure. Members of the ATP-binding cassette (ABC) family mediate drug efflux, while solute carrier (SLC) transporters facilitate drug uptake. Inherited variation in these transporters contributes to inter-individual differences in chemotherapy pharmacokinetics and toxicity, although clinical translation remains less standardized than for enzymatic pathways [26,27].

Finally, germline variation in DNA repair pathways influences how normal tissues respond to chemotherapy-induced DNA damage. Differences in repair capacity modulate susceptibility to hematologic toxicity, mucosal injury, and secondary malignancies, further reinforcing the importance of host genetics in shaping treatment tolerance [5,12].

### 4.3. Clinical Evidence and Guideline-Supported Applications

The clinical utility of pharmacogenetics in oncology is supported by a growing body of evidence and formal guideline recommendations. Organizations such as the Clinical Pharmacogenetics Implementation Consortium (CPIC) and the Dutch Pharmacogenetic Working Group (DPWG) provide genotype-based dosing guidelines for multiple chemotherapeutic agents, including fluoropyrimidines, thiopurines, and irinotecan [11,25].

Regulatory agencies increasingly recognize the relevance of PGx. Both the FDA and EMA include pharmacogenetic information in drug labeling, and the EMA actively recommends pre-treatment DPYD testing prior to fluoropyrimidine therapy. These regulatory endorsements reflect the strong association between genotype-informed dosing and reduced incidence of severe toxicity [11,13].

Real-world implementation studies demonstrate that PGx-guided prescribing can significantly reduce chemotherapy-related hospitalizations, treatment delays, and dose-limiting toxicities, without compromising antitumor efficacy. However, uptake remains variable across institutions and healthcare systems, highlighting the gap between evidence generation and routine clinical practice [5,21].

### 4.4. Limitations as a Standalone Tool

Despite its proven value, pharmacogenetics has inherent limitations when applied in isolation. Most notably, PGx primarily informs treatment safety rather than efficacy. A patient with favorable pharmacogenetic markers may tolerate chemotherapy well but still derive little or no therapeutic benefit if the tumor is intrinsically resistant [25,26].

Moreover, germline PGx provides no tumor-specific information. It describes host drug handling but does not capture somatic alterations, transcriptional states, or microenvironmental factors that determine tumor sensitivity. As such, PGx cannot substitute for tumor molecular profiling but must be integrated with somatic and functional data to achieve true precision oncology [2,16].

Finally, clinical adoption remains inconsistent, hindered by barriers such as limited physician education, upfront testing costs, turnaround time constraints, and heterogeneous reimbursement policies. Addressing these challenges will require coordinated efforts in clinician training, infrastructure development, and health policy reform [13,21].

Therefore, within the broader molecular oncodiagnostic paradigm outlined in this review, pharmacogenetics occupies a patient-centric safety and dosing axis, complementing tumor transcriptomics and functional response assays. Its greatest clinical value emerges not as a standalone predictor, but as an essential component of an integrated strategy aimed at maximizing therapeutic benefit while minimizing avoidable harm.

## 5. Integrating Tumor Transcriptomics and Patient Pharmacogenetics

The convergence of tumor transcriptomics and patient pharmacogenetics (PGx) represents a critical evolution of precision oncology, addressing the long-standing disconnect between tumor-centered molecular stratification and host-specific determinants of drug handling. As outlined in earlier sections, approaches focused exclusively on either tumor biology or patient genetics fail to fully explain variability in chemotherapy response. Transcriptomic profiling captures the functional state and adaptive potential of malignant cells, whereas germline PGx defines the pharmacological landscape within which anticancer agents operate. Their integration establishes a dual-perspective framework that more accurately constrains predictions of therapeutic benefit, toxicity risk, and overall treatment feasibility.

### 5.1. Biological Rationale for Integration

Effective chemotherapy requires the simultaneous alignment of two biologically distinct but interdependent conditions. The first is tumor susceptibility, which is governed by transcriptional programs controlling proliferation, DNA repair capacity, apoptosis, drug metabolism, and interactions with the tumor microenvironment. Gene expression profiling provides a dynamic functional readout of pathway activity that frequently surpasses static genomic alterations in predicting drug sensitivity or adaptive resistance [3,8,28].

The second condition is patient tolerability, determined by inherited variation in genes involved in absorption, distribution, metabolism, and excretion (ADME), as well as by host tissue susceptibility to cytotoxic injury [11,25]. Germline PGx profiling captures these determinants, enabling anticipation of systemic exposure, toxicity risk, and dose-limiting adverse events.

Considering either dimension in isolation leads to biologically incomplete decision-making. A transcriptomically sensitive tumor may be clinically untreatable at effective doses due to host toxicity, while a pharmacogenetically favorable patient may derive no benefit from a regimen to which the tumor exhibits transcriptional resistance. This asymmetry underscores the necessity of integrated diagnostics. Tumor transcriptomics primarily informs whether a drug should work by identifying activated oncogenic pathways, compensatory resistance mechanisms, and microenvironmental constraints [2,9]. In contrast, PGx informs whether a drug can be safely administered, guiding dose optimization and toxicity avoidance without providing tumor-specific efficacy insights [5,13]. Integration reconciles these dimensions, reframing chemotherapy selection as a biologically constrained optimization problem rather than a probabilistic extrapolation.

### 5.2. Multi-Omics Integration Strategies

Building on this rationale, multi-omics integration strategies seek to unify inherited pharmacokinetic predispositions with real-time tumor biology. The conceptual emergence of multi-omics approaches reflects a broader recognition that complex biological systems, including cancer, cannot be fully understood through single-layer molecular analysis alone [29,30]. Composite biomarkers that combine germline DNA variants with tumor RNA expression profiles have demonstrated superior predictive performance compared with isolated genomic or transcriptomic markers [30,31]. By jointly modeling host drug handling and tumor-intrinsic vulnerability, these approaches generate unified response scores that more closely reflect clinical outcomes.

Transcriptome-informed risk stratification has already demonstrated clinical utility in selected diagnostic assays, while PGx-guided dosing has reduced severe adverse drug reactions for fluoropyrimidines, thiopurines, and irinotecan. Their combination enables simultaneous optimization of efficacy and safety, a goal that neither modality can achieve independently [11,26].

Systems biology approaches provide a conceptual scaffold for integration by modeling drug–gene–pathway interactions across both tumor and host compartments. Network-based frameworks map chemotherapeutic agents onto tumor signaling networks while incorporating host metabolic enzymes and transporters, enabling simulation of patient-specific therapeutic trajectories [23,28]. These models reflect a shift from descriptive biomarkers toward predictive systems medicine, emphasizing interaction rather than single-variable association.

Advanced machine learning and deep learning architectures further facilitate multi-omic integration. Transformer-based encoders and attention-driven fusion models can accommodate high-dimensional RNA expression data alongside germline variant information, generating unified predictive outputs for response and toxicity [30,32]. While many such models remain preclinical, their validation in large public datasets, including The Cancer Genome Atlas (TCGA), supports the feasibility of scalable integrative oncodiagnostics [16,31].

### 5.3. Emerging Evidence from Recent Studies

Recent studies published between 2024 and 2025 consistently indicate that integrated multi-omics models outperform single-omics predictors in forecasting treatment response, progression-free survival, and adverse drug events. Transcriptome-based precision oncology platforms have demonstrated clinical utility across heterogeneous and treatment-resistant malignancies, particularly when combined with patient-specific host variables [9,10]. Multi-omics analyses further uncover latent resistance drivers and compensatory signaling pathways that are not apparent from genomic data alone [31,33].

Validation efforts leveraging TCGA and other large consortia datasets confirm that integrated models capture clinically relevant heterogeneity overlooked by single-layer analyses [2,31]. Collectively, these findings reinforce the concept that drug response emerges from interacting tumor and host systems rather than from isolated molecular events.

### 5.4. Clinical Decision-Making Implications

Integrated oncodiagnostics provide a framework for moving beyond standardized regimens toward biologically informed protocol selection that balances tumor vulnerability with patient safety. This approach supports the rational exclusion of treatments predicted to be ineffective or intolerable, reducing unnecessary toxicity and therapeutic delay [4,16].

Incorporating PGx-guided dosing into transcriptomically informed treatment selection allows proactive adjustment of drug exposure to remain within therapeutic windows, an especially relevant consideration for agents with narrow therapeutic indices [5,21].

Finally, integrated models facilitate risk avoidance by identifying patients unlikely to benefit from specific chemotherapies due to either intrinsic tumor resistance or host metabolic vulnerability. These insights support treatment de-escalation and the rational selection of alternative strategies, including targeted therapies, immunotherapy, or functional precision approaches such as ex vivo drug testing, which are discussed in subsequent sections [14,34].

## 6. Ex Vivo Chemoresistance Testing Using Patient-Derived Tumor Cells

Ex vivo chemoresistance testing using patient-derived tumor cells has transitioned from a historical research interest into a high-stakes component of functional precision oncology (FPO). Traditional reliance on genomic and transcriptomic profiling for therapeutic decision-making, while transformative, often fails to capture the complex, emergent properties of drug response that arise from tumor architecture and microenvironmental interactions. Functional assays using living tumor material bridge this gap by providing real-time, direct measurement of tumor chemoresistance, thereby offering a complementary axis of biological information to molecular profiling [14,35]. Throughout this section, technologies are discussed according to their current stage of development, ranging from research-grade platforms to pilot clinical implementations. Only a limited subset has reached formal clinical validation or reimbursement, and most remain investigational despite encouraging feasibility data.

### 6.1. Current Technological Landscape (2026)

The landscape of ex vivo chemoresistance testing in 2026 is defined by advances in model systems, assay platforms, and computational integration. Patient-derived models have become increasingly robust, with studies confirming high success rates in establishing high-grade glioma (HGG) organoids (>90%), although certain molecular subtypes (e.g., IDH1-mutant tumors) remain comparatively refractory to culture [36].

A driving rationale for 3D model systems such as organoids and multicellular spheroids (MCSs) is their capacity to recapitulate key features of in vivo tumor biology—including hypoxia gradients, cell–cell and cell–matrix interactions, metabolic zonation, and heterogeneous proliferative states—that are lost in two-dimensional culture. Multicellular spheroids, in particular, have been extensively characterized as scalable and reproducible platforms for modeling solid tumor architecture and drug penetration dynamics, and are increasingly proposed as intermediate systems bridging traditional in vitro assays and patient-specific therapeutic testing. These properties support their growing role in preclinical drug development and in exploratory personalized drug susceptibility screening [34,37,38].

Recent breakthroughs in digital twin technology—computational frameworks that integrate patient-specific data to simulate tumor behavior—have shown promise in brain cancer, enabling virtual treatment simulations that predict emergent resistance patterns prior to clinical administration. Such digital twins synthesize multi-modal data including ex vivo drug responses, transcriptomics, and metabolic flux estimates, providing an unprecedented level of personalized therapeutic forecasting [39].

Parallel advances in assay platforms such as the VitroScan 3D micro-tumor testing system have been highlighted in early 2026 as providing actionable, ex vivo functional insights by interrogating drug efficacy across a range of micro-tumor constructs. These high-throughput platforms streamline the transition from biopsy to phenotypic drug response output, significantly reducing turnaround times and enhancing clinical feasibility [14].

### 6.2. Clinical Utility and Feasibility

The clinical utility of ex vivo chemoresistance assays is increasingly supported by prospective and interventional studies across diverse cancer types and treatment contexts. In hematologic malignancies, the SMARTrial (NCT03488641) demonstrated that ex vivo drug response profiling can be translated into clinically relevant timelines, with drug response reports delivered within 7 days for over 90% of participants. Importantly, ex vivo resistance to standard chemotherapeutic agents correlated with treatment failure, highlighting the predictive value of functional phenotyping beyond genomic markers alone [40,41].

In pediatric oncology, ongoing trials (e.g., NCT03860376) aim to combine high-throughput drug sensitivity testing (DST) with genomic profiling to guide individualized therapy. Preliminary feasibility data suggest that integration of ex vivo DST can inform therapeutic selection in treatment-resistant pediatric cancers, where molecular predictors alone often underperform [42].

Functional testing is also catalyzing drug repositioning efforts, identifying unexpected efficacy of existing compounds in specific cancer contexts. For example, high-content screening platforms have revealed selective inhibitory effects of the VEGF inhibitor axitinib in BCR-ABL1-driven leukemias, a finding that could prompt re-evaluation of off-label utility in subgroups defined by functional response rather than canonical genomic signatures.

### 6.3. Emerging Enhancements

A key frontier in ex vivo chemoresistance testing is the integration of machine learning and high-content data. Composite pathway scores that fuse drug-induced transcriptomic changes with predictive algorithms are emerging as mechanistically interpretable biomarkers of sensitivity, exemplified by studies on agents such as QAL333, where integrated transcriptomic–bioinformatic models delineate determinants of selective cytotoxicity [43].

Single-cell level interrogation of drug response dynamics, although still nascent in oncology relative to bacterial systems like Antimicrobial Single-Cell Testing (ASCT), is poised to transform phenotypic profiling. Techniques that quantify killing dynamics and heterogeneity at single-cell resolution hold potential to deconvolve resistant subpopulations that drive relapse, merging with single-cell transcriptomic approaches that have already revealed heterogeneous microenvironmental states linked to chemotherapy resistance [20,44].

Beyond technology, efforts to democratize functional testing are underway. The establishment in 2025 of the first-in-Africa ex vivo drug sensitivity testing platform underscores the importance of expanding infrastructure to diverse patient cohorts. Early results from South African leukemia samples not only validate assay performance but also identify novel drug combinations tailored to cohort-specific response patterns, highlighting that regional expansion of functional testing can unmask population-specific therapeutic signals with direct clinical relevance [45].

Ex vivo chemoresistance testing has re-emerged as a promising and increasingly investigated component of FPO, supported by advances in 3D culture systems and assay miniaturization. Through innovations in 3D culture systems, digital twin modeling, high-throughput phenotypic platforms, and integrative computational biology, functional assays now provide actionable insights that complement genomic and transcriptomic data. The expanding evidence base—from hematologic malignancies to pediatric cancer trials and global platform deployment—indicates both clinical feasibility and potential to reshape individualized cancer therapy. However, its clinical implementation remains largely confined to research settings and pilot clinical programs, with heterogeneous levels of validation across tumor types. Key challenges include model interpretability, data harmonization, computational cost, and the absence of prospective clinical outcome validation (Table 1).

## 7. Emerging Predictive Technologies and Computational Frameworks in Precision Oncology

Advances in molecular oncology have generated a diverse ecosystem of technologies aimed at improving prediction of therapeutic response and disease evolution. These approaches range from clinically established tools, such as companion diagnostics and circulating biomarker assays, to emerging computational frameworks that integrate multi-omic data with artificial intelligence-based predictive modeling. Importantly, these technologies differ substantially in their current level of clinical maturity and translational readiness. While some are already incorporated into routine diagnostic workflows, others remain primarily exploratory and are still undergoing methodological refinement and prospective validation. The following section therefore reviews this spectrum of predictive technologies, highlighting both their potential clinical utility and the limitations that currently constrain their broader implementation in precision oncology.

In addition to traditional biomarker-driven approaches, emerging diagnostic strategies increasingly incorporate computational modeling and artificial intelligence to integrate complex molecular and functional datasets. The progressive incorporation of molecular diagnostics into oncology has reshaped clinical decision-making, moving cancer care beyond histopathological classification toward biologically informed stratification. Building on the integrative framework outlined in previous sections, contemporary precision oncology platforms increasingly rely on genomic, transcriptomic, and multi-omic data to inform therapeutic selection and risk stratification [16]. Artificial intelligence (AI) and machine learning (ML) methods serve as critical interpretative layers within this ecosystem, facilitating the extraction of clinically relevant patterns from high-dimensional molecular datasets [23].

Whereas earlier sections focused on the biological rationale and diagnostic foundations of integrated oncodiagnostics, this section addresses how complex molecular data are interpreted, prioritized, and translated into clinical decisions, as well as the role of AI in supporting these processes within real-world healthcare settings.

### 7.1. From Molecular Findings to Clinical Decisions

The clinical utility of molecular oncodiagnostics lies not solely in the detection of genetic or transcriptional alterations, but in their contextual interpretation within disease biology, therapeutic options, and patient-specific factors. Somatic mutations, copy number alterations, gene fusions, and transcriptomic signatures increasingly inform diagnosis, prognosis, and treatment selection across multiple tumor types [4,13].

Molecular results rarely function as deterministic signals. Instead, they contribute probabilistic evidence that must be weighed alongside clinicopathological features, disease stage, and treatment history. Integrated profiling strategies—combining genomic and transcriptomic layers—enhance interpretability by capturing functional consequences of molecular alterations, including pathway activation states, co-occurring aberrations, and intratumoral heterogeneity [31,46].

This interpretative complexity underpins the continued importance of multidisciplinary molecular tumor boards. Within these settings, oncologists, molecular pathologists, geneticists, and bioinformaticians collaboratively contextualize molecular findings and align them with therapeutic strategies. Such frameworks improve both variant interpretation and clinical decision-making, particularly when results are ambiguous or biologically complex [21].

### 7.2. Companion Diagnostics and Precision Therapeutics

The co-development of targeted therapies and companion diagnostics (CDx) represents a tangible clinical success of molecular oncodiagnostics. Companion diagnostics are assays developed alongside therapeutic agents to identify patients most likely to benefit from treatment or to avoid harm. Regulatory approval of many modern anticancer drugs now depends on the demonstration of predictive biomarker engagement or clinical utility within defined molecular subgroups, effectively linking diagnostic testing to therapeutic decision-making [47,48].

This paradigm extends beyond classical kinase–inhibitor pairings to encompass a broad spectrum of genomic alterations, structural rearrangements, and expression-based signatures. Integrative molecular profiling enables detection of both dominant oncogenic drivers and subtler modulators of drug response, supporting refined patient stratification even in the absence of single actionable mutations. Transcriptome-based platforms further enhance therapy alignment across heterogeneous and treatment-resistant malignancies, underscoring the value of multi-omic integration in clinical practice [9,16].

Established clinical examples illustrate this principle. HER2 testing in breast and gastric cancer guides the use of HER2-targeted therapies [48,49], while EGFR mutation and ALK rearrangement testing direct tyrosine kinase inhibitor use in non-small cell lung cancer [50,51]. Detection of BRAF V600E mutations in melanoma determines eligibility for BRAF/MEK inhibitor combinations, and KRAS mutation status in colorectal cancer excludes patients from anti-EGFR antibody therapy [50]. Additional biomarkers, including BRCA1/2 and PD-L1, further exemplify the breadth of CDx-guided therapeutic decision-making [48,49].

Despite these advances, important challenges persist. Tumor evolution under therapeutic pressure can yield residual or recurrent disease with molecular profiles distinct from the original diagnostic specimen, limiting the predictive value of baseline testing unless longitudinal monitoring strategies are employed [52]. Moreover, the rapid expansion of actionable targets has exceeded the capacity of single-analyte assays, necessitating comprehensive genomic and transcriptomic platforms that generate complex result sets requiring advanced bioinformatic and multidisciplinary interpretation [9,16]. Regulatory considerations, including analytical validation and cross-platform reproducibility, are increasingly relevant as multiplex and AI-assisted classifiers replace traditional assays.

### 7.3. Liquid Biopsy in Clinical Monitoring

Liquid biopsy technologies, particularly circulating tumor DNA (ctDNA) analysis, have emerged as valuable complements to tissue-based diagnostics. Their minimally invasive nature enables serial sampling, facilitating dynamic monitoring of tumor burden, clonal evolution, and emerging resistance mechanisms [16].

In specific clinical scenarios, liquid biopsies provide actionable insights when tissue sampling is infeasible or insufficient. However, interpretation requires careful consideration of biological and technical limitations. Low tumor fraction, clonal hematopoiesis, and pre-analytical variability can confound results, particularly in early-stage disease or minimal residual disease settings [21]. Consequently, liquid biopsy findings are most informative when integrated with tissue-based data, imaging, and clinical context rather than used in isolation.

### 7.4. Ethical, Regulatory, and Health System Considerations

The expanding application of molecular oncodiagnostics raises ethical and regulatory challenges beyond analytical performance. Incidental germline findings, management of variants with uncertain significance, and disparities in access to advanced diagnostics require careful governance to ensure equitable implementation [21].

From a health system perspective, clinical utility and cost-effectiveness remain central concerns. While comprehensive molecular profiling can improve outcomes in selected populations, indiscriminate testing without clear therapeutic implications risks increasing healthcare expenditure without proportional benefit. Evidence-based guidelines and outcome-linked reimbursement frameworks are therefore essential for sustainable integration of advanced diagnostics into routine oncology care [16].

### 7.5. Emerging Trends and Future Perspectives

Molecular oncodiagnostics is moving toward an integrative framework that combines genomic, transcriptomic, epigenomic, and proteomic data. The inclusion of additional regulatory layers, such as post-translational modifications, is increasingly recognized as essential for understanding disease mechanisms and therapeutic response in complex biological systems. This multi-omics approach enhances tumor characterization and supports more precise therapeutic stratification [31,53].

AI and ML approaches are anticipated to further support pattern recognition, variant interpretation, and outcome prediction, although transparency, robustness, and prospective validation remain prerequisites for broader clinical adoption [23,54]. Emerging technologies such as single-cell sequencing and spatial profiling promise deeper insight into intratumoral heterogeneity and tumor–microenvironment interactions, reinforcing the need for adaptive regulatory and clinical frameworks capable of accommodating rapid technological evolution [46].

Among emerging computational approaches, the concept of digital tumor twins has recently attracted attention as a potential framework for integrating multi-omic, imaging, and clinical data into dynamic patient-specific models of disease behavior. In principle, digital twins aim to simulate tumor evolution and therapeutic responses by integrating biological knowledge with machine-learning or mechanistic modeling strategies. These models function as computational counterparts of individual patients in which genomic, transcriptomic, imaging, and clinical datasets are combined to explore potential treatment trajectories and optimize therapeutic decision-making. In this sense, digital twins have been proposed as a possible extension of precision oncology, complementing molecular diagnostics by enabling in silico evaluation of therapeutic hypotheses before clinical implementation [55,56].

Despite this conceptual appeal, the current clinical maturity of digital tumor twins and related AI-driven predictive models remains limited. Many existing implementations rely on retrospective datasets, simplified biological assumptions, or highly curated training cohorts, which may limit their generalizability to real-world clinical settings. Consequently, most digital twin frameworks should presently be regarded as exploratory research tools that support hypothesis generation and model development rather than clinically validated decision-support systems. Recent reviews of digital twin applications in oncology similarly emphasize that substantial methodological, computational, and validation challenges remain before these models can be reliably translated into routine clinical practice [57,58].

Importantly, predictive modeling enhances interpretation and prioritization of molecular data but remains complementary to the biological and functional validation strategies discussed in the subsequent section.

## 8. Functional Integration of Multi-Omic and Ex Vivo Data

**Figure 2:**
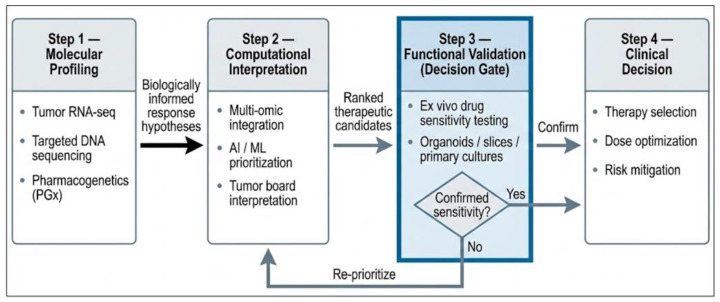
Stepwise workflow for integrating molecular profiling, predictive modeling and ex vivo functional validation in precision oncology. The figure illustrates the progression from molecular and pharmacogenetic profiling through computational integration and AI-assisted prioritization to ex vivo functional validation as a decision gate, culminating in informed clinical treatment selection and optimization.

Building on the molecular rationale outlined in Section 5 and the interpretative frameworks discussed in Section 7, a central translational challenge in precision oncology is the reconciliation of predictive models with the actual behavior of tumors under therapeutic pressure. The integrative oncodiagnostic framework discussed here is intended as a conceptual synthesis rather than a new technological platform. While many functional precision oncology approaches focus primarily on tumor-derived drug sensitivity assays, the present framework emphasizes the structured integration of three complementary information layers: tumor regulatory state (captured through transcriptomics), host pharmacological constraints (defined by germline pharmacogenetics), and phenotypic drug response measured through ex vivo testing platforms. Aligning these distinct data streams within a single decision-support workflow enables molecular hypotheses regarding drug response to be evaluated through direct functional testing. In this model, molecular profiling generates biologically informed predictions, whereas ex vivo assays provide phenotypic validation of whether predicted vulnerabilities translate into measurable suppression of tumor viability. Importantly, this approach should be regarded as a translational framework currently explored in research and pilot clinical programs rather than as a standardized clinical workflow (Figure 2).

Placed within a systems biology context, functional integration acknowledges that tumor behavior emerges from coordinated interactions among genomic, transcriptomic, epigenomic, and metabolic networks. When interpreted together and subjected to functional challenge, multi-omic data provide a more constrained and biologically grounded basis for evaluating therapeutic susceptibility than molecular profiling alone [59,60].

### 8.1. Informing Drug Response Expectations with Multi-Omic Data

At the predictive stage, multi-omic integration aims to construct a systems-level expectation of drug response by contextualizing molecular alterations within regulatory and metabolic programs active in the tumor and host. Transcriptomic profiling, most commonly via RNA sequencing, reveals differentially expressed genes and coordinated regulatory modules that are not apparent from DNA sequence analysis alone. These transcriptional states frequently reflect compensatory signaling pathways, altered apoptotic thresholds, or efflux mechanisms that modulate sensitivity to therapy.

Network-based analyses, including those integrating single-cell RNA sequencing data with regulatory maps, have elucidated resistance mechanisms by identifying transcriptional modules and upstream effectors associated with diminished drug responsiveness [61]. More broadly, coherent expression programs predictive of pathway engagement demonstrate that baseline transcriptional states can encode functional dependencies relevant to therapeutic susceptibility [62].

Pharmacogenetic profiling contributes a complementary, patient-centric dimension by informing systemic drug handling. Variants in genes encoding drug-metabolizing enzymes and transporters influence systemic exposure, intracellular drug availability, and toxicity risk. Although PGx does not directly determine tumor cell kill, it constrains the feasibility of achieving effective drug concentrations in vivo and informs dose modulation strategies aimed at balancing efficacy and safety [63].

Despite the depth of information provided by genomic, transcriptomic, and pharmacogenetic data, molecular predictions remain probabilistic. Nominally actionable alterations do not uniformly confer drug sensitivity across biological contexts, as broader transcriptional and signaling landscapes can attenuate or bypass oncogenic dependencies. Multi-omic integration therefore refines predictive expectations by embedding genomic findings within functional regulatory states and host-specific pharmacological constraints [59].

### 8.2. From Multi-Omic Prediction to Ex Vivo Validation

To translate molecular predictions into actionable evidence, integrative precision oncology frameworks increasingly incorporate ex vivo functional assays as a downstream validation step. In this graded workflow, hypotheses generated from multi-omic profiling are subjected to direct pharmacological challenge using viable tumor tissue, patient-derived organoids (PDOs), or slice cultures. These assays aim to provide near-real-time phenotypic readouts of drug response, confirming or refuting molecularly inferred sensitivities.

Importantly, this integrative strategy is not standardized for routine clinical use and remains confined to translational research settings and selected pilot precision oncology initiatives. Nevertheless, it offers a biologically grounded mechanism for testing whether predicted vulnerabilities translate into measurable suppression of tumor viability under controlled conditions.

Table 2 summarizes the integrative workflow proposed in this review, illustrating how multiple biological and analytical layers converge to support individualized therapeutic decision-making in precision oncology. The framework begins with molecular characterization of the tumor genome, which identifies oncogenic drivers and potential therapeutic targets. This information is complemented by tumor transcriptomic profiling, which captures dynamic pathway activity and regulatory states that influence drug sensitivity and resistance. In parallel, germline pharmacogenetic analysis provides patient-specific insight into drug metabolism and toxicity risk, thereby constraining the therapeutic window for candidate treatments. These molecular and host-derived data streams can then be integrated through computational modeling and artificial intelligence-assisted approaches, enabling prioritization of therapeutic strategies within a systems-level context. At the downstream stage, ex vivo functional assays—including PDOs and tumor slice cultures—serve as phenotypic validation platforms that directly test whether predicted vulnerabilities translate into measurable suppression of tumor viability. Finally, the integration of molecular predictions and functional evidence occurs within multidisciplinary molecular tumor boards, where clinical expertise contextualizes diagnostic findings and informs individualized treatment selection.

Although conceptually coherent, this integrative framework remains largely translational. The different components exhibit heterogeneous levels of clinical maturity: genomic and transcriptomic profiling are increasingly embedded in clinical diagnostics, pharmacogenetics is selectively implemented according to guideline-supported indications, whereas ex vivo functional testing and multi-omic predictive modeling remain primarily confined to research settings and pilot precision oncology programs.

### 8.3. Functional Validation and Refinement of Therapeutic Strategies

Within this framework, ex vivo functional profiling serves several interrelated roles. First, it functions as a confirmatory layer, testing whether predicted dependencies derived from multi-omic analysis manifest as measurable reductions in tumor viability. Studies in gastric cancer employing ex vivo drug testing on endoscopic samples illustrate how phenotypic assays can validate—or challenge—molecular predictions, occasionally revealing sensitivities not anticipated from molecular signatures alone [64].

Second, ex vivo profiling refines therapeutic prioritization when multi-omic analyses suggest multiple plausible targets. In pediatric precision oncology, systematic ex vivo drug sensitivity testing has complemented molecular profiling to rank candidate therapies, revealing differential responses among compounds predicted to be effective based on omic features alone [65]. This refinement is particularly valuable in highly heterogeneous tumors, where subclonal diversity may obscure single-target dependencies.

Third, integration of drug-induced transcriptional responses enables identification of dynamic vulnerability signatures—functional states that emerge only after pharmacological challenge. Frameworks capturing such response modules have demonstrated improved capacity to classify tumors according to mechanism-based sensitivity, bridging the gap between static molecular profiles and dynamic treatment response [62].

Collectively, these roles underscore that functional validation is not merely confirmatory but iterative. Multi-omic profiling generates mechanistic hypotheses, ex vivo assays test and contextualize those hypotheses, and the resulting data inform subsequent interpretative models. This feedback loop exemplifies a learning precision oncology paradigm that integrates molecular richness with empirical validation.

Functional integration of multi-omic and ex vivo data has progressed from an aspirational concept toward a practical translational foundation for precision oncology. By aligning molecular predictions with phenotypic reality, this approach reconciles computational modeling with biological complexity. Nevertheless, several methodological and translational limitations currently constrain the clinical deployment of ex vivo drug sensitivity testing. Establishment success rates vary substantially across tumor types, with organoid cultures derived from certain epithelial malignancies demonstrating higher reproducibility than those from highly stromal or necrotic tumors. In addition, culture conditions may incompletely reproduce tumor microenvironmental factors such as immune interactions, vascular dynamics, and systemic pharmacokinetics, which can influence therapeutic response in vivo.

The predictive accuracy of organoid-based drug testing has shown encouraging results in several early clinical studies, with reported concordance between ex vivo sensitivity and patient response often exceeding 70–80% in selected cohorts [66,67]. However, most evidence derives from retrospective analyses or small prospective studies, and standardized assay protocols, response metrics, and inter-laboratory reproducibility remain active areas of investigation. Large-scale prospective validation studies and harmonized experimental frameworks will therefore be essential to establish ex vivo functional testing as a robust clinical biomarker capable of guiding routine therapeutic decision-making.

Early clinical implementation studies are beginning to explore the feasibility of integrating multi-omic profiling with functional precision oncology platforms. Prospective precision medicine initiatives incorporating transcriptomic or multi-omic tumor profiling have demonstrated that layered molecular information can increase the proportion of patients matched to targeted therapies compared with genomic profiling alone [68]. More recent translational precision oncology programs integrating multiple molecular layers have further demonstrated the practical feasibility of multi-omics-guided therapeutic decision-making in clinical settings [69]. Complementary clinical analyses from the same research framework have also indicated that multi-omics-guided therapy can be implemented safely in advanced cancer patients, supporting the translational viability of complex molecular profiling strategies [70].

In parallel, functional precision oncology studies using patient-derived organoids or ex vivo drug-sensitivity testing have shown encouraging concordance between experimental drug responses and clinical outcomes in selected patient cohorts, supporting the potential of phenotype-guided therapy prioritization [66,67]. More recent feasibility studies further suggest that organoid-based platforms may help inform treatment decisions in refractory malignancies such as pancreatic cancer, highlighting the translational potential of integrating functional assays with molecular profiling strategies [71,72].

**Figure 3:**
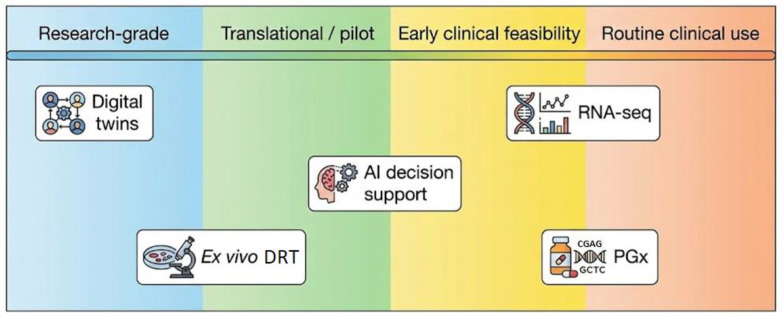
Clinical readiness spectrum of functional precision oncology technologies. The figure illustrates the progression from research-grade and translational tools (e.g., digital twins and ex vivo drug response testing) to early clinical feasibility and routine clinical use (e.g., RNA sequencing and pharmacogenetics), highlighting differences in technological maturity and clinical implementation. Technologies differ substantially in maturity and regulatory status; none currently constitute fully standardized, reimbursed diagnostic workflows.

Although these studies remain limited in scale and heterogeneity, they provide early proof-of-concept that integrated molecular and functional frameworks may improve therapeutic matching and inform the design of future prospective precision oncology trials. At present, functional integration should be regarded as an advanced decision-support strategy under active investigation rather than an established standard of care (Figure 3).

## 9. Clinical Implementation: Opportunities and Barriers

The translation of ex vivo functional testing—particularly using PDOs—into routine clinical oncology represents a profound opportunity to bridge gaps left by static genomic or pathological predictions. By 2026, PDOs and related ex vivo platforms have matured from research tools into clinically relevant assays capable of informing therapeutic choices when standard molecular diagnostics are equivocal or insufficient. Nonetheless, the path toward widespread clinical implementation remains encumbered by logistical, temporal, economic, and regulatory barriers that must be addressed to realize the full promise of functional precision medicine.

### 9.1. Translation to Clinical Practice

In many clinical scenarios, molecular sequencing fails to identify a clear actionable mutation, or multiple drugs appear equally viable based on genomic or transcriptomic profiles alone. In such “equivocal” cases, ex vivo testing has emerged as a functional biomarker to guide drug selection. PDOs and other ex vivo systems provide a phenotypic readout of tumor sensitivity to drugs, effectively serving as a patient avatar against which therapy can be tested before administration.

These ex vivo pipelines are increasingly structured around systematic evaluation of tumor sensitivity to panels of clinically approved agents and synergistic drug combinations. Studies implementing PDOs in advanced colorectal cancer demonstrate that organoid chemosensitivity profiles can meaningfully stratify responses to standard therapies, highlighting potential avenues for personalized selection beyond genomic indication alone [13,72,73]. Importantly, more recent clinical evidence in metastatic colorectal cancer further supports substantial concordance between PDO-based predictions and observed treatment outcomes, reinforcing the potential of ex vivo assays as clinically informative predictive tools rather than exploratory platforms alone [74].

Importantly, real-world evidence suggests substantial concordance between ex vivo predictions and clinical outcomes. In both metastatic gastrointestinal cancers and hematologic malignancies, ex vivo drug responses have shown high predictive power, with response profiles correlating with clinical benefit in a majority of cases. For example, comprehensive ex vivo drug sensitivity profiling in hematologic cancer cohorts has demonstrated that functional response patterns align with treatment effectiveness and clinical outcomes, underscoring the potential for PDOs to serve as true predictive assays rather than exploratory research tools [40,75].

In acute myeloid leukemia (AML), where genetic markers alone often fail to reliably predict response to targeted inhibitors, PDO-based models have been used as patient avatars to anticipate therapeutic failure and to identify alternative effective agents, offering proof-of-principle for ex vivo guidance in aggressive hematologic contexts [40].

Despite their promise, the operational realities of ex vivo functional testing present significant obstacles to timely clinical application. A primary concern is turnaround time (TAT). Typical workflows for establishing viable PDO cultures, expanding them to sufficient numbers, and completing comprehensive “chemograms” reporting drug sensitivity require a median of approximately six weeks, with observed ranges extending from four to ten weeks. While this timeline may be acceptable for guiding second-line or adjuvant therapy decisions, it remains too slow to inform first-line treatment in patients with rapidly progressing disease, where immediate decisions are imperative. The delay is further compounded by the necessity of maintaining sample viability and functional integrity across multiple culture steps [76].

Another critical factor is logistical constraint: PDO establishment success varies significantly with tissue type and the nature of the biopsy. For instance, core needle biopsies—commonly used for minimally invasive sampling—exhibit a PDO take-on rate that is far from optimal, often near 60–65%, meaning a sizable fraction of samples fail to yield usable organoids for drug testing. Because PDO growth is inherently dependent on tissue quality and immediate processing, maintaining tight control over pre-analytical variables—such as ischemia time and sample transport conditions—is essential to maximize culture success [77].

Scalability and cost remain persistent hurdles. By early 2020s estimates, functional precision medicine workflows incorporating ex vivo testing were associated with high per-patient costs—often approaching hundreds of thousands of dollars in resource-intensive environments like North America—a barrier that has tempered widespread adoption in routine practice. Although technological developments (e.g., robotic liquid-handling systems and automated culture platforms) are beginning to improve throughput and reproducibility, cost models must improve markedly to support scalability across diverse healthcare systems and patient populations by 2027 and beyond [76].

Moreover, automated systems must be integrated thoughtfully to ensure that the gains in efficiency do not come at the expense of data quality or biological fidelity. The transition from bespoke, laboratory-specific PDO models to standardized, reproducible clinical assays will require significant investment in infrastructure and training, as well as continuous evaluation of quality control protocols.

### 9.2. Ethical, Regulatory, and Health System Challenges

The regulatory landscape for integrating ex vivo functional data into clinical decision-making is evolving, but remains incompletely defined. Traditional drug approval pathways and clinical guideline frameworks have been structured around randomized controlled trials and well-established biomarkers. By contrast, the use of functional data from ex vivo models—particularly in rare cancers or subgroups where traditional trials are impractical—requires novel evidence frameworks that balance scientific rigor with practical utility.

Beyond regulatory validation, reimbursement policies represent a critical determinant of clinical adoption. Many functional precision oncology platforms, including patient-derived organoid assays and multi-omic profiling pipelines, remain outside established reimbursement frameworks in most healthcare systems. Current reimbursement models are typically designed for single diagnostic tests linked to specific therapeutic indications, whereas integrative oncodiagnostic approaches combine multiple analytical layers and computational interpretation. The absence of clear reimbursement pathways may therefore limit implementation to well-resourced academic centers, even when clinical utility is demonstrated. Health technology assessment frameworks capable of evaluating multi-modal diagnostic strategies will be required to determine cost-effectiveness and support broader clinical integration.

As of 2026, regulatory agencies such as the U.S. Food and Drug Administration (FDA) and the European Medicines Agency (EMA) are actively evaluating pathways for incorporating “external” data—including ex vivo and real-world evidence—to support label expansions or therapeutic recommendations in contexts where controlled trial data may be limited or unobtainable [73]. These discussions include assessments of the analytical validity, clinical validity, and clinical utility of PDO-based assays, as well as consideration of how best to integrate complex datasets from multiple sources (including AI-assisted interpretation) without compromising patient safety.

Equitable access to advanced oncodiagnostic technologies represents an additional ethical consideration. Comprehensive molecular profiling, computational analysis, and ex vivo functional testing require specialized infrastructure that is currently concentrated in high-income healthcare systems and major academic centers. This concentration risks widening existing disparities in cancer care if precision oncology tools remain inaccessible to patients treated in community settings or resource-limited regions. Efforts to standardize protocols, develop scalable platforms, and support international research collaborations will therefore be essential to ensure that emerging functional precision medicine strategies do not inadvertently exacerbate global inequities in cancer diagnosis and treatment.

Another emerging regulatory focus centers on artificial intelligence and machine learning systems used to interpret ex vivo data. The interpretive “opacity” of certain AI models has drawn scrutiny, prompting standards development around validation, documentation, and continuous monitoring of AI-enabled decision-support tools. Regulatory frameworks increasingly emphasize transparency and explainability as prerequisites for clinical deployment, adding a layer of complexity for vendors and healthcare institutions seeking to leverage advanced analytics within functional precision medicine platforms [78]. 

For ex vivo testing to be widely accepted as a standard diagnostic rather than a research adjunct, robust clinical validation in prospective, multi-center contexts is essential. Non-interventional trials like the SMARTrial have already demonstrated feasibility: in hematologic malignancies, drug response reports were successfully delivered within seven days for 91% of participants, meeting primary feasibility endpoints and underscoring the potential for rapid functional phenotyping to inform clinical decisions [40].

However, standardization remains a critical concern. Variability in culture media, extracellular matrices (such as Matrigel), and other batch-dependent reagents can introduce confounding effects that obscure true biological sensitivity. Regulatory bodies emphasize the need for harmonized protocols and cross-site quality assessments to ensure that ex vivo results are reproducible, comparable, and interpretable across institutions [79].

Beyond regulatory and technical challenges, ethical issues loom large in the clinical implementation of PDO-based testing. Functional biobanks—repositories of living tumor models linked to clinical, molecular, and outcome data—are powerful resources for both clinical decision support and research. Nevertheless, their governance raises questions about informed consent, ownership of derivative data, and the rights of patients to withdraw consent once samples are processed.

Ensuring that consent forms encompass the potential secondary uses of patient-derived tissues—including longitudinal data linkage and sharing with external research partners—is imperative to uphold patient autonomy and trust. Additionally, the use of living tissues in large-scale biobanks intersects with broader concerns about data privacy and the equitable return of benefit, particularly when proprietary analytics or commercial partnerships are involved.

The clinical implementation of ex vivo testing—especially using PDOs—stands at the nexus of promise and complexity. These functional assays offer a pathway to individualized therapy selection when standard molecular predictors fall short, particularly in cancers with heterogeneous or cryptic response determinants. High concordance between ex vivo sensitivity profiles and clinical outcomes in multiple cancer types highlights the potential of PDOs as actionable functional biomarkers. Yet logistical realities—including turnaround time, tissue viability, cost, and scalability—temper immediate clinical uptake.

Regulatory developments offer cautious optimism, with agencies exploring new evidence paradigms that integrate functional data and advanced analytics into clinical decision-making. Prospective validation, harmonization of protocols, and robust ethical frameworks for consent and biobanking are essential to support this transition. As these barriers are addressed, the integration of ex vivo data into precision oncology practice will likely accelerate, helping to fulfill the long-held vision of truly individualized cancer therapy.

## 10. Final Considerations

### 10.1. Value Added by Functional Testing

Functional chemosensitivity assays, particularly those based on ex vivo culture systems such as PDOs and tissue slices, bring a distinct and clinically valuable dimension to precision oncology by providing direct evidence of drug activity against patient-specific tumor cells. Unlike static genomic or transcriptomic profiles, which infer potential drug sensitivity based on molecular features, functional tests measure actual tumor cell responses to therapeutic agents before these are administered to the patient. This direct readout enables clinicians to exclude therapies that are unlikely to be effective, thereby sparing patients from unnecessary toxicity, the emotional burden of ineffective treatment, and the economic costs associated with failed therapies [80].

Another hallmark of contemporary functional assays is their ability to capture biological complexity that eludes purely molecular data. While genomic sequencing identifies mutations and transcriptomics delineates expression patterns, these approaches do not fully recapitulate the dynamic interplay between tumor cells and their microenvironment. Advanced ex vivo platforms—including organotypic tumor slices and three-dimensional organoid cultures—maintain key elements of tissue architecture, extracellular matrix interactions, and stromal support, all of which can modulate drug response. These features are particularly relevant for therapies targeting pathways influenced by cell–cell and cell–matrix signaling, which are poorly represented in two-dimensional cell lines or sequence data alone [81].

The clinical feasibility of ex vivo functional profiling has been demonstrated in prospective studies. Most notably, the SMARTrial validated the integration of ex vivo drug response profiling into hematologic oncology practice. In this non-interventional trial, functional chemosensitivity assays were able to generate clinically interpretable drug response reports within seven days for approximately 91% of participants, establishing a proof of concept that phenotypic data can be delivered on timescales compatible with real-world clinical decision-making [40]. This achievement highlights the translational potential of functional platforms as rapid decision support tools rather than exclusively as research assays.

Taken together, these observations underscore the added value of functional testing in precision oncology: functional data can validate, refine, and, in some cases, override genomic predictions, offering a patient-specific functional axis to guide therapy selection.

### 10.2. Integration Challenges

Despite their promise, several significant challenges limit the full integration of functional testing into routine clinical workflows.

A primary barrier is the variable success in establishing viable cultures from clinical specimens. Different ex vivo models exhibit differing “take” rates; for example, tissue slice assays and PDOs often succeed in a majority of samples, but failures are not uncommon. Reported success rates for some organotypic slice assays hover around 77%, with technical failures frequently attributed to high baseline apoptosis, sample contamination, or inadequate tissue quality. This variability underscores the need for rigorous pre-analytical quality control and optimized protocols to maximize the yield of functional material from biopsies and surgical samples [82].

Another critical hurdle relates to reproducibility across laboratories. Functional assays, particularly those requiring long-term cultures or sensitive read-outs, are influenced by the details of culture conditions, choice of viability assay (e.g., ATP-based luminescence vs. dye-exclusion), and handling procedures. Without harmonized standard operating procedures (SOPs), inter-laboratory variability can confound interpretation and diminish confidence in cross-study comparisons. Although high technical repeatability has been demonstrated in controlled settings and short-term assays, longer-term culture systems (>1 year) often exhibit greater variability, emphasizing the necessity of standardized reagents, matrices, and analytical approaches [82,83].

A further challenge lies in linking functional assay results with hard clinical endpoints such as overall survival or progression-free survival at scale. While retrospective studies show strong correlations between functional assay results and clinical response, recent evidence from large cohorts demonstrates this predictive capability in modern practice. In AML patients, ex vivo chemosensitivity profiles generated by the PharmaFlow platform were strongly associated with complete remission rates and trends in overall survival, highlighting that functional testing can meaningfully forecast clinical outcomes [84]. Modern functional ex vivo assays have shown promising early clinical correlations: ex vivo sensitivity tests adapted for biopsy-sized breast cancer tissues demonstrate high feasibility and technical success across multiple cytotoxic agents [85], and in a proof-of-concept study, anthracycline-based ex vivo sensitivity predictions were concordant with in vivo MRI responses in approximately 75% of patients receiving neoadjuvant chemotherapy [86].

### 10.3. Future Directions

Looking forward, the field is poised for several transformative developments that aim to surmount the current limitations and broaden clinical impact.

Future research is increasingly directed toward prospective, multi-center clinical trials that integrate transcriptomics, pharmacogenetics, and ex vivo chemosensitivity data into unified precision medicine platforms. Trials such as pediatric drug sensitivity testing (DST) guided studies and large consortia (e.g., the proposed EXALT framework) exemplify efforts to evaluate multi-modal predictive models that synergize molecular and functional insights. Such integrative study designs promise to refine predictive algorithms, identify novel biomarkers of response, and validate composite signatures with stronger prognostic and predictive power than any single modality alone [81].

Development of standardized, clinically validated functional platforms represents a critical frontier in precision oncology. Among the most promising technological advances are cancer-on-chip microphysiological systems—microfluidic platforms that recreate essential aspects of the tumor microenvironment, including three-dimensional architecture, biochemical gradients, stromal interactions, and perfused vasculature. These devices offer a substantially enhanced capacity to model tumor biology and interrogate therapeutic responses in a context that more closely mirrors patient physiology than traditional culture systems [87,88]. These platforms enable higher-throughput drug screening while accommodating limited biopsy material. They also support granular functional phenotyping through automated high-content imaging that quantifies dynamic and spatially resolved responses. When microphysiological platforms are combined with robotic culture handling and advanced imaging analytics, the potential arises to reduce operator-dependent variability and accelerate functional profiling, making it increasingly feasible to incorporate phenotype data into routine clinical timelines and decision-support workflows [89,90].

Large-scale research initiatives are also contributing to the translation of these functional platforms into clinical practice. Collaborative programs that invest in cross-disciplinary research teams aim to tackle some of the most complex challenges in cancer biology and therapeutic implementation. These initiatives provide sustained support for international, cross-disciplinary collaboration. As a result, they facilitate the development and validation of innovative precision medicine technologies, including standardized cancer-on-chip systems and automated functional assays aligned with clinical and regulatory requirements. These efforts bring together clinicians, basic scientists, bioengineers, and regulatory experts to ensure that emerging technologies are developed with translational relevance and prospective clinical application in mind, paving the way for functional precision medicine to move beyond isolated research settings into broader clinical use [87,88,89].

The evolution of functional chemosensitivity testing represents a critical advance in the ongoing quest to personalize cancer therapy. By providing direct therapeutic evidence, capturing biological complexity beyond static molecular read-outs, and demonstrating clinical feasibility in prospective settings such as the SMARTrial, functional assays have carved out a unique and indispensable niche within the precision oncology paradigm.

Yet the integration of functional testing into mainstream practice is not without obstacles. Variability in culture establishment, challenges in standardization, and the need for robust correlation with long-term clinical outcomes highlight the complexities inherent in translating innovative science into dependable clinical tools.

Future progress hinges on well-designed integrative clinical trials, standardized and scalable technologies, and collaborative implementation initiatives that can move functional assays from specialized research environments into widely accepted clinical diagnostics. As these developments unfold, the convergence of multi-omic and functional data promises to enrich precision medicine, offering clinicians and patients more reliable, individualized treatment guidance.

## 11. Conclusions

The evolving convergence of molecular profiling and functional phenotyping represents a fundamental shift in how precision oncology is practiced. Molecular diagnostics, including genomics and transcriptomics, have equipped clinicians with powerful tools to characterize cancer at the level of DNA, RNA, and regulatory networks. Yet these static measurements alone are often insufficient to capture the dynamic and adaptive nature of tumor behavior under therapeutic pressure. Functional assays directly measure tumor cell responses outside the patient, particularly through patient-derived organoids and ex vivo tissue cultures. These systems provide crucial evidence of drug activity and can validate or refine predictions derived from molecular data.

Functional testing adds value across several dimensions: it offers direct therapeutic evidence that can exclude ineffective drugs and help avoid unnecessary toxicity; it captures biological complexity that reflects tumor microenvironment interactions absent from genomic profiles; and it has been shown to be feasible within clinically relevant timeframes. These features position functional assays as a critical complement to molecular signatures in guiding treatment decisions, particularly when genomic evidence is ambiguous or multiple therapeutic options exist.

Despite this potential, substantial challenges remain. Variability in culture success rates, differences in assay protocols across laboratories, and the need for correlation with long-term clinical outcomes underscore the importance of methodological standardization and validation in broader cohorts. Furthermore, economic and logistical considerations—including turnaround time, tissue requirements, and operational costs—currently limit the clinical uptake of ex vivo functional testing. Addressing these barriers will require sustained innovation in assay technology, automated and scalable platforms, and thoughtful integration with clinical workflows. At present, these workflows should be regarded as advanced decision-support strategies under active evaluation rather than validated standards of care.

Looking ahead, the future of precision oncology lies in the seamless integration of multi-omic data with functional phenotyping, guided by rigorous prospective studies and supported by regulatory frameworks attuned to complex, multi-dimensional evidence. Beyond technical validation, successful clinical translation will also require the development of sustainable reimbursement models and policies that ensure equitable access to advanced oncodiagnostic technologies across diverse healthcare systems. Cross-disciplinary collaborations will be essential to advance functional platforms from research settings into everyday clinical practice. As these efforts progress, the synergy between molecular insight and functional reality promises to enable more accurate, individualized therapy selection, ultimately improving patient outcomes and fulfilling the promise of truly personalized cancer care.

## Figures and Tables

**Table 1:** Current developmental stage and clinical adoption of selected functional precision oncology technologies (2026), distinguishing research-grade tools from pilot or early clinical feasibility implementations. None represent standardized, routine clinical diagnostics.

Technology	Current Status (2026)	Clinical Use
PDO drug sensitivity testing	Pilot clinical feasibility	Select centers/trials
Tumor slice cultures	Research-pilot	Limited
Digital twins	Research-grade	Experimental
High-throughput DST platforms	Early clinical feasibility	Non-routine

**Table 2:** Integrative framework for multi-omic and functional oncodiagnostics in precision oncology. The table summarizes the sequential workflow linking tumor molecular characterization, transcriptomic profiling, patient pharmacogenetics, computational integration, and ex vivo functional validation to support individualized therapeutic decision-making. The framework highlights the complementary roles of molecular prediction and phenotypic testing within a translational precision oncology pipeline.

Stage	Data Type	Functional Role	Clinical Output
Molecular characterization	Tumor genomics	Identifies driver alterations and therapeutic targets	Targeted therapy eligibility
Functional state analysis	Tumor transcriptomics	Defines pathway activation and resistance programs	Prediction of chemotherapy sensitivity
Host pharmacology	Germline pharmacogenetics	Predicts drug metabolism and toxicity risk	Dose adjustment/treatment safety
Computational integration	Multi-omic modeling/AI	Integrates tumor and host data to prioritize therapies	Ranked treatment options
Functional validation	Ex vivo drug testing (PDOs, slices)	Direct measurement of tumor drug sensitivity	Confirmation of therapeutic efficacy
Clinical decision	Multidisciplinary tumor board	Integration of molecular and functional evidence	Individualized treatment selection

## Data Availability

No new data were created or analyzed in this study.
